# UAC-Net: a unified multi-task network with aggregated contextual attention and cross-layer fusion for brain tumor segmentation and classification

**DOI:** 10.3389/fnins.2026.1986322

**Published:** 2026-10-20

**Authors:** Chuanqi Cai, Jincan Zhang, Dongyan Zhang, Wenna Chen, Xinghua Tan, Ganqin Du, Hongwei Jiang

**Affiliations:** 1 College of Information Engineering, Henan University of Science and Technology, Luoyang, China; 2 The First Affiliated Hospital, and College of Clinical Medicine of Henan University of Science and Technology, Luoyang, China

**Keywords:** ACA, ALMFF, brain tumor, multi-task learning, UAC-Net

## Abstract

**Introduction:**

Accurate segmentation and classification of brain tumors from magnetic resonance imaging (MRI) are critical for clinical diagnosis and treatment planning. However, existing methods typically treat these two tasks independently, failing to exploit their inherent complementarity, and often struggle to capture multi-scale contextual information essential for identifying small or irregular lesions.

**Methods:**

To address these limitations, this paper proposes UAC-Net, a unified multi-task learning framework built upon a U-Net backbone that jointly optimizes tumor segmentation and classification through a shared encoder. Two core modules are introduced: (1) the Aggregated Contextual Attention (ACA) module, which enhances dynamic perception of critical lesion regions by combining local sliding-window attention with global downsampling attention across all encoder levels; and (2) the Adjacent Layer Multi-scale Feature Fusion (ALMFF) module, which enables cross-level feature interaction to enrich multi-scale representations. In the segmentation branch, ALMFF-fused features are progressively upsampled to achieve precise lesion localization; in the classification branch, ACA-enhanced deep features are aggregated via global average pooling to yield robust semantic representations for tumor typing.

**Results:**

Experiments on two publicly available brain tumor MRI datasets demonstrate that UAC-Net achieves competitive and consistent performance compared with several representative methods. Specifically, UAC-Net obtains a classification accuracy of 98.53% and a Dice coefficient of 81.39% on the Figshare dataset, and achieves 99.07% classification accuracy and 86.61% Dice coefficient on the BRISC 2025 dataset.

**Discussion:**

These findings suggest that integrating contextual attention and cross-layer feature fusion within a unified multi-task framework is a promising approach for joint brain tumor segmentation and classification.

## 1 Introduction

Brain tumors, including meningiomas, gliomas, and pituitary tumors, are among the most critical neurological disorders threatening human health and pose substantial challenges for clinical diagnosis and therapeutic decision-making (McFaline-Figueroa and Lee, 2018). Magnetic resonance imaging (MRI), owing to its superior soft-tissue contrast, has become an important imaging modality for brain tumor evaluation. Accurate identification of tumor types and precise delineation of lesion regions from MRI are essential for computer-aided diagnosis, treatment planning, and disease assessment.

Early computer-aided brain tumor analysis primarily relied on conventional image-processing techniques, such as thresholding, region growing, edge detection, clustering, and morphological operations (Bibi et al., 2025). These methods can be effective under relatively controlled imaging conditions, but their performance is often sensitive to image intensity variations, noise, and the considerable heterogeneity in tumor appearance. In addition, many of these approaches depend heavily on manually designed rules and prior knowledge, which limits their adaptability across different imaging conditions (Mohsen et al., 2026).

To overcome these limitations, machine-learning-based approaches were subsequently introduced. Handcrafted features describing intensity, texture, shape, and statistical characteristics were combined with classifiers such as support vector machines, random forests, and k-nearest-neighbor algorithms for brain tumor detection, classification, and segmentation (Dorfner et al., 2025). Compared with conventional rule-based methods, these approaches improved the ability to model complex image patterns. However, their performance remained highly dependent on the quality and discriminative capability of manually engineered features.

The emergence of deep learning further reduced the reliance on handcrafted feature engineering by enabling hierarchical representations to be learned directly from medical images in an end-to-end manner (Shoushtari et al., 2025). Convolutional neural networks (CNNs) have demonstrated strong feature representation capabilities in brain tumor classification, while encoder-decoder architectures, particularly U-Net and its variants, have been widely adopted for tumor segmentation (Yang et al., 2025). More recently, attention mechanisms and Transformer-based architectures have been introduced to improve lesion localization, multi-scale feature representation, and long-range contextual modeling (Abueed et al., 2025).

Despite the substantial progress achieved by deep learning-based methods in brain tumor image analysis (Lu et al., 2025), several challenges remain unresolved. First, brain tumors often occupy relatively small regions in MRI images and exhibit considerable variations in shape, size, and location, making accurate lesion localization difficult (R et al., 2025). Second, conventional convolution-based models are effective in extracting local features but may be limited in capturing long-range contextual dependencies, which are important for identifying irregular tumor structures (Yang et al., 2025). Third, many existing methods treat segmentation and classification as two independent tasks, thereby overlooking the potential complementarity between pixel-level structural information and image-level semantic information (Lv et al., 2025; Rhanoui et al., 2025). Segmentation provides fine-grained spatial information about tumor regions, whereas classification provides high-level semantic information related to tumor categories. Effectively exploiting the complementary information between these two tasks may therefore improve both lesion localization and tumor discrimination.

To address these challenges, this paper proposes UAC-Net, a unified multi-task learning framework for joint brain tumor segmentation and classification. The proposed network employs a shared encoder to learn common feature representations for the two tasks while using task-specific branches for segmentation and classification. To enhance cross-level feature interaction, an Adjacent Layer Multi-scale Feature Fusion (ALMFF) module is introduced to integrate spatial details and semantic information from adjacent encoder stages. In addition, an Aggregated Contextual Attention (ACA) module is incorporated to combine local sliding-window attention with global spatial-downsampling attention, thereby strengthening both local lesion representation and long-range contextual modeling. Through joint multi-task optimization, UAC-Net exploits the complementary relationship between pixel-level structural information and image-level semantic information to improve brain tumor segmentation and classification simultaneously. The main contributions of this paper are summarized as follows:

- A unified multi-task learning framework, UAC-Net, is proposed for joint brain tumor segmentation and classification. By sharing encoder representations between the two tasks, the framework exploits fine-grained structural information from segmentation to aid classification, thereby facilitating collaborative feature learning and joint optimization.
- An Adjacent Layer Multi-scale Feature Fusion (ALMFF) module is designed to enhance cross-level feature interaction. The module integrates features from adjacent encoder stages and employs multi-scale convolutional branches to enrich spatial details and semantic representations, thereby improving feature representation for downstream segmentation and classification tasks.
- An Aggregated Contextual Attention (ACA) module is introduced to strengthen contextual modeling. By combining local sliding-window attention with global spatial-downsampling attention, together with learnable query embeddings and positional modeling, ACA captures both fine-grained lesion information and long-range dependencies, thereby improving the representation of small and irregular tumor regions.

## 2 Related work

Deep learning methods based on CNNs have been widely applied in the field of medical image analysis, achieving significant progress particularly in brain tumor classification and segmentation tasks (Kanzawa et al., 2024).

Representative classification networks, including AlexNet (Saladi et al., 2025) and its improved variants, as well as DenseNet and its derivatives (Mao et al., 2025), have demonstrated favorable performance in feature extraction. In segmentation tasks, U-Net and its derived models (R et al., 2025), leveraging encoder-decoder architectures and skip connection mechanisms, have achieved effective integration of low-level spatial details and high-level semantic information, greatly advancing the development of medical image segmentation (Jha et al., 2020; Xiao et al., 2018). To further improve feature fusion between the encoder and decoder, U-Net++++ introduced nested and dense skip pathways to reduce the semantic gap between features at different levels (Zhou et al., 2018). However, such methods inherently rely on local receptive fields, making it difficult to effectively capture long-range contextual dependencies. In brain tumor MRI images, where lesion regions are typically small and morphologically irregular, this limitation further constrains the model’s ability to extract critical discriminative features.

To enhance feature extraction capability while controlling computational complexity, attention mechanisms have been widely incorporated into CNN models. For example, Attention U-Net integrates attention gates into the U-Net architecture to suppress irrelevant background responses and emphasize task-relevant regions, thereby improving lesion localization (Schlemper et al., 2019). Based on the dimension of focus, existing methods can be broadly categorized into channel attention, spatial attention, and multi-scale attention. Channel attention mechanisms enhance the representation of key semantic information by adaptively weighting different feature channels (Huang et al., 2021). Spatial attention focuses on localizing critical regions within images, guiding the network to attend to lesion locations (Mazumdar and Mukherjee, 2022). Multi-scale attention further integrates contextual information across different scales to improve adaptability to targets with varying morphologies (Gao et al., 2025). These attention mechanisms effectively guide the network to focus on critical lesion regions, thereby improving segmentation accuracy.

Although the introduction of attention mechanisms has, to some extent, enhanced the capability of CNNs to perceive critical regions, they remain inherently constrained by the local receptive field of convolution operations, making it difficult to effectively model global contextual information. In recent years, the Transformer architecture has garnered widespread attention in the field of computer vision due to its powerful global modeling capability (Han et al., 2023). Vision Transformer (ViT) and its variants have demonstrated superior performance when trained on large-scale datasets. In the medical imaging domain, Transformer-based architectures have increasingly been introduced into segmentation frameworks to enhance global contextual modeling. TransUNet combines CNN-based local feature extraction with Transformer-based long-range dependency modeling, thereby integrating local spatial details and global contextual information for medical image segmentation (Chen et al., 2024). Swin-Unet further adopts a hierarchical shifted-window Transformer within a U-shaped architecture to model multi-scale features while reducing the computational burden of global self-attention (Cao et al., 2023). However, Transformer-based methods still exhibit notable limitations. The quadratic complexity of the self-attention mechanism incurs substantial computational and memory overhead. Although local attention (Liu et al., 2022) and spatial downsampling strategies (Wang et al., 2022) have alleviated this issue to some extent, the former restricts the receptive field, while the latter results in the loss of fine-grained feature representation. Moreover, the multi-layer stacking architecture may suffer from insufficient information interaction due to residual degradation issues.

Although convolutional neural networks, attention mechanisms, and Transformer architectures have all achieved significant progress in the field of medical image analysis, existing methods still commonly treat segmentation and classification as independent tasks, overlooking the intrinsic relationship between them (Li et al., 2023). However, in brain tumor image analysis, the pixel-level structural information targeted by segmentation tasks and the image-level semantic information required by classification tasks are inherently highly complementary–precise lesion segmentation facilitates the extraction of more discriminative classification features, while accurate classification results can in turn guide segmentation to focus on critical regions (Shi, 2024).

In recent years, some medical image studies have begun exploring multi-task learning frameworks for segmentation and classification. For instance, joint training of the two tasks has been achieved by introducing a classification branch into U-Net (Chen et al., 2019; He et al., 2020), or through joint optimization strategies that leverage complementary information across tasks to enhance overall performance (Ling et al., 2024). These approaches have, to some extent, demonstrated the feasibility and effectiveness of multi-task learning. However, existing frameworks still lack effective cross-scale feature interaction mechanisms and contextual modeling capabilities, making it difficult to fully capture the deep correlations between segmentation and classification tasks, thereby limiting further performance improvement (Zhou et al., 2023).

Overall, existing CNN-based, attention-based, Transformer-based, and multi-task learning methods have promoted the development of brain tumor image analysis. However, several limitations still remain. First, CNN-based models are effective in local feature extraction but may be insufficient in modeling long-range contextual dependencies. Second, Transformer-based methods improve global representation but often introduce high computational complexity and may require large-scale data for stable training. Third, existing multi-task frameworks still lack effective cross-scale feature interaction and contextual enhancement mechanisms, which limits their ability to fully exploit the complementary relationship between segmentation and classification tasks. These limitations motivate further research on unified frameworks that can jointly enhance lesion localization and tumor type discrimination.

## 3 Proposed method

### 3.1 Network structure of UAC-Net

UAC-Net adopts a U-Net architecture consisting of shared layers and task-specific layers, forming an end-to-end network architecture as illustrated in Figure 1. The shared layer consists of encoder convolutional blocks, while the task-specific layers include classification and segmentation subnetworks. Instead of employing simple concatenation in the skip connections, the model first fuses features from adjacent downsampling layers within the U-Net architecture through an ALMFF module to extract common features that capture cross-level and multi-scale information. Subsequently, an aggregated contextual attention module is applied to enhance the contextual information of the features.

**FIGURE 1:**
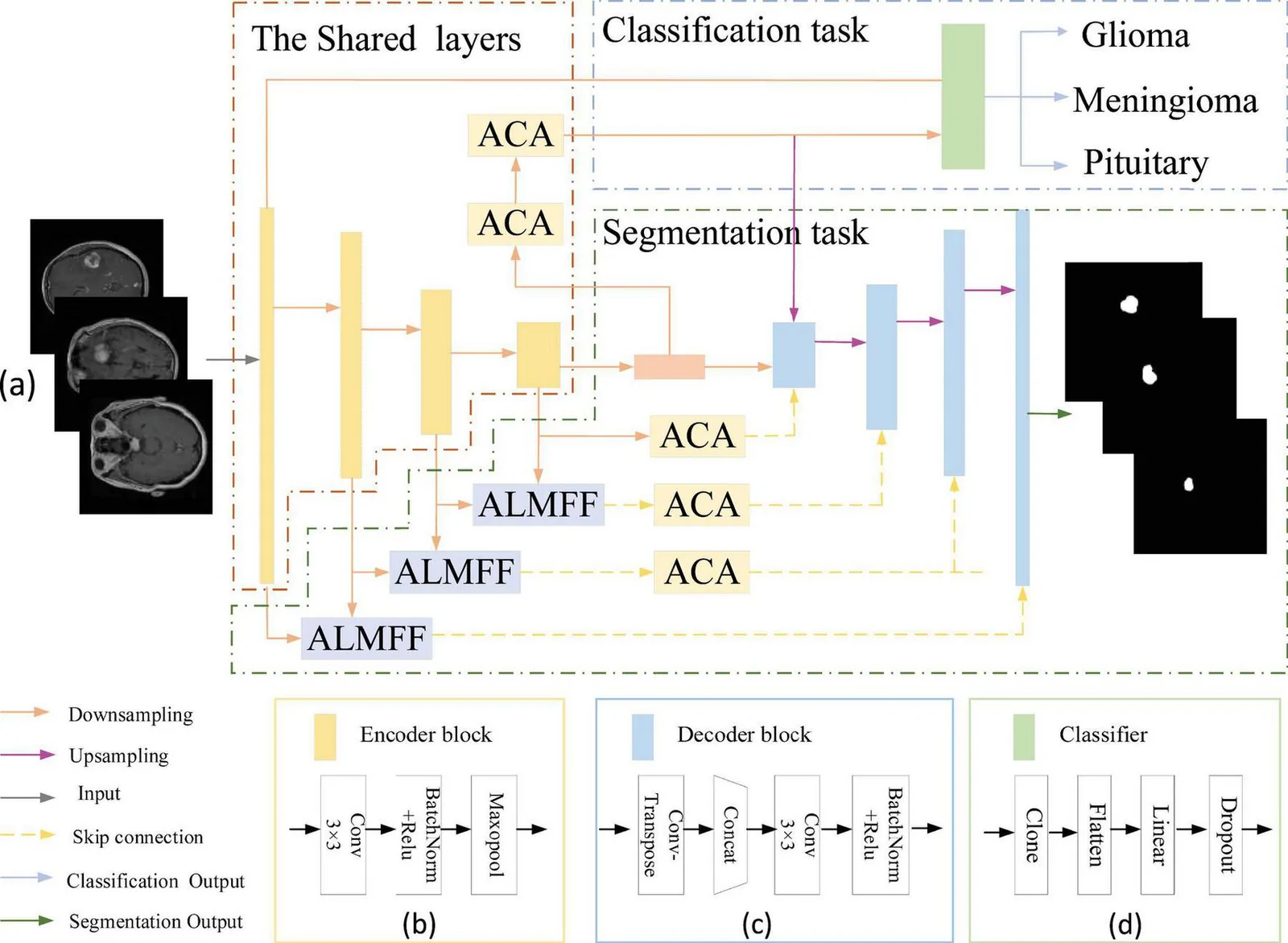
The network architecture of the proposed UAC-Net. **(a)** The overall network architecture, including shared layers, segmentation task, and classification task; **(b)** the encoder block; **(c)** the decoder block; **(d)** the classification branch for tumor prediction.

Specifically, the input image sequentially passes through four downsampling blocks, progressively extracting multi-scale feature maps while compressing spatial resolution, thereby forming a shared encoder. In the segmentation branch, the ALMFF module performs interactive fusion of features from adjacent layers to generate cross-level, multi-scale feature representations. Subsequently, the fused features are enhanced by the ACA module and combined with progressive upsampling in the decoder to gradually restore spatial resolution, ultimately producing the segmentation mask. In the classification branch, the deepest ACA-enhanced features are processed through GAP (Global Average Pooling, GAP) to obtain a global semantic vector, which is then mapped via fully connected layers to classification probabilities for three tumor types. The two branches share the same encoder features, achieving complementary enhancement between pixel-level structure and image-level semantics through joint multi-task optimization.

### 3.2 ALMFF

Although the skip connections in U-Net can transmit shallow spatial details to the decoder, directly concatenating encoder and decoder features may introduce a semantic gap between low-level texture information and high-level semantic representations. This problem is particularly prominent in brain tumor MRI analysis, where tumor regions are often small, irregular in shape, and ambiguous along their boundaries. Shallow features preserve fine-grained edge and location information, whereas deeper features contain stronger semantic cues but lose part of the spatial details due to repeated downsampling. Therefore, simply using single-level features or directly concatenating features from different stages may be insufficient for accurate lesion localization. To address this issue, we design the Adjacent Layer Multi-scale Feature Fusion (ALMFF) module to promote effective interaction between adjacent encoder layers and generate more discriminative multi-scale representations for the subsequent segmentation and classification branches. Figure 2 illustrates its architecture. Specifically, let the adjacent-level features be denoted as *f*_*i*_ ∈ ℝ^*C* × *H* × *W*^, *f*_*i*+1_ ∈ ℝ^2*C* ×  0.5*H* ×  0.5*W*^respectively. First, the deep feature *f*_*i*+1_is adjusted via transposed convolution to obtain $f_{i+1}^{\prime }\in {\mathbb{R}}^{C\times H\times W}$, which can be expressed as shown in Equation 1:


$$f_{i+1}^{\prime }=C\,o\,n\,v\,T\,r\,a\,n\,s\,p\,o\,s\,e\,\left(f_{i+1}\right)$$ (1)

**FIGURE 2:**
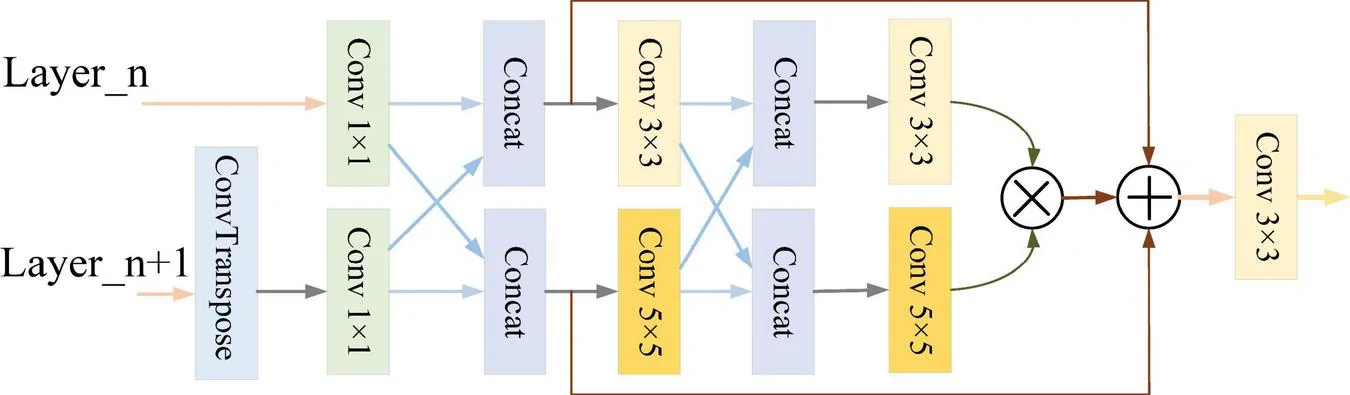
The detailed architecture of ALMFF.

Then, fusion is performed using a dual-branch structure. The 3 × 3 convolution is used to capture local spatial details and boundary-related information, while the 5 × 5 convolution enlarges the receptive field to introduce broader contextual cues. The convolved features are then fused through element-wise multiplication, which enhances consistent responses between adjacent layers and suppresses redundant background information. Finally, a residual connection is introduced to preserve the original information and stabilize feature propagation, yielding the output of the module as described in Equation 2:


$$\begin{array}{c} f_{i}^{\prime }=C\,o\,n\,v_{3\times 3}\,\left(C\,o\,n\,c\,a\,t\,\left(f_{i},f_{i+1}^{\prime }\right)\right)\\ f_{i+1}^{{\prime \prime }^{}}=C\,o\,n\,v_{5\times 5}\,\left(C\,o\,n\,c\,a\,t\,\left(f_{i+1}^{\prime },f_{i}\right)\right)\\ f_{o\,u\,t}=\left(f_{i}^{\prime }\times f_{i+1}^{{\prime \prime }^{}}\right)+f_{i}+f_{i+1}^{{\prime \prime }^{}} \end{array}$$ (2)

### 3.3 ACA

ACA is an attention module inspired by biological vision, simulating foveal vision and continuous eye movements. As shown in Figure 3, through a dual-path design combining sliding window and spatial downsampling, the module enables each token to simultaneously capture local details and global contextual information. In addition, the module introduces learnable query embeddings to enhance the flexibility of attention computation, positional attention to provide dynamic positional bias, and length-scaled cosine attention to stabilize attention weights across varying input lengths via a learnable scaling factor.

**FIGURE 3:**
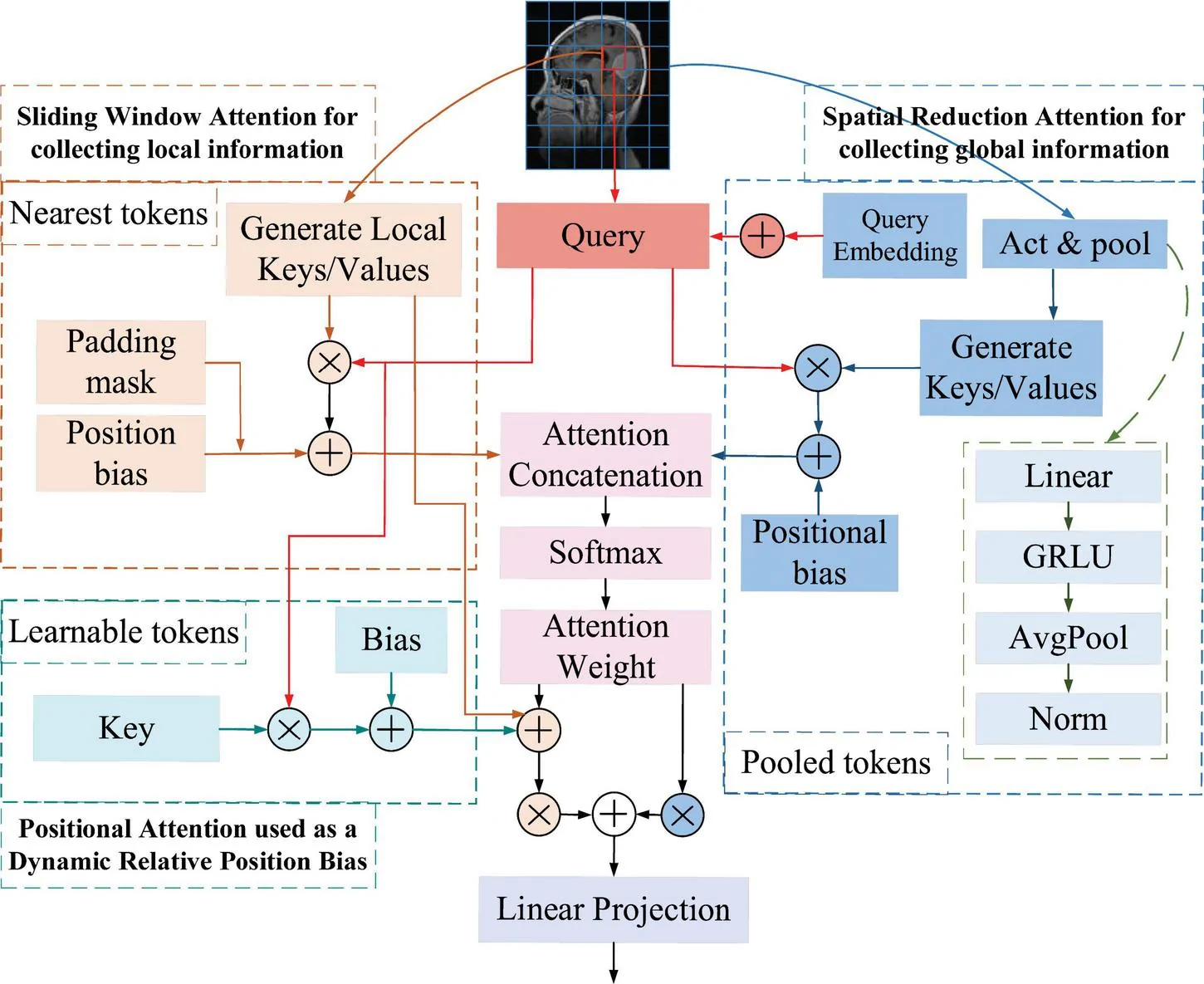
The detailed architecture of ACA.

Given an input feature map *X* ∈ *R*^*C* × *H* × *W*^, let each pixel position be denoted as (*i*, *j*). The set of pixels within a sliding window of size *kÂt’* is defined as *ρ*_*(i,j)*_, where *ρ*_*(i,j)*_ = *k*^2^. The global pixel set obtained via pooling operations is denoted as σ(*x*), with a pooling size of *H*_*p*_ × *W*_*p*_, yielding σ(*x*) = *H*_*p*_ × *W*_*p*_. The sliding window attention and spatial downsampling attention are computed as shown in Equation 3:


$$\begin{array}{c} S_{\rho \,\left(i,j\right)}=Q_{\left(i,j\right)}\,K_{\rho_{\left(i,j\right)}}^{T}\\ S_{\sigma \,\left(i,j\right)}=Q_{\left(i,j\right)}\,K_{\sigma_{\left(X\right)}}^{T} \end{array}$$ (3)

where *Q*_(*i*,*j*)_ denotes the query vector, *K*_*ρ*(*i,j*)_ represents the key vectors corresponding to pixels within the sliding window, and *K*_σ(*x*)_ denotes the global key vectors obtained via pooling operations. The attention weights are computed as shown in Equation 4:


$$A_{\left(i,j\right)}=s\,o\,f\,t\,\max\left(\frac{C\,o\,n\,c\,a\,t\,\left(S_{\rho \,\left(i,j\right)},S_{\sigma \,\left(i,j\right)}\right)}{\sqrt{d}}+B_{\left(i,j\right)}\right)$$ (4)

The output is computed as shown in Equation 5:


$$P\,F\,A_{\left(i,j\right)}=A_{\rho \,\left(i,j\right)}\,V_{\rho \,\left(i,j\right)}+A_{\sigma \,\left(i,j\right)}\,V_{\sigma \,\left(X\right)}$$ (5)

To further enhance the model’s information aggregation capability, learnable query embeddings and a position attention mechanism are introduced. These mechanisms allow the model to generate a dynamic affinity matrix without relying on input queries, thereby enabling more flexible attention computation, as shown in Equation 6:


$$\begin{array}{c} S_{\rho \,\left(i,j\right)}=\left(Q_{\left(i,j\right)}+Q\,E\right)\,K_{\rho_{\left(i,j\right)}}^{T}\\ S_{\sigma \,\left(i,j\right)}=\left(Q_{\left(i,j\right)}+Q\,E\right)\,K_{\sigma_{\left(X\right)}}^{T} \end{array}$$ (6)

where, the *QE* is a learnable query embedding vector designed to enhance the representational capacity of the query vector. The position attention can be expressed as shown in Equation 7:


$$P\,F\,A\,\left(X_{\left(i,j\right)}\right)=\left(A_{\rho \,\left(i,j\right)}+Q_{\left(i,j\right)}\,T\right)\,V_{\rho \,\left(i,j\right)}+A_{\sigma \,\left(i,j\right)}\,V_{\sigma \,\left(X\right)}$$ (7)

here, *T* denotes the learnable position attention matrix, which introduces dynamic positional bias. The length-scaled cosine attention mechanism is designed to address the variation of attention weights under multi-scale inputs. By introducing a learnable length-scaling cosine factor, this mechanism ensures consistent output confidence of the attention mechanism across varying input lengths, as formulated in Equation 8:


$$A\,t\,t\,e\,n\,t\,i\,o\,n\,\left(Q,K,V\right)=s\,o\,f\,t\,\max\left(\tau \,\log N\cdot \hat{Q}\,{\hat{K}}^{T}\right)\,V$$ (8)

where $\hat{Q}$and $\hat{K}$are the normalized query and key vectors, τ is a learnable scaling factor, and *N* denotes the number of interactions between each query and valid key vectors. The aggregated contextual attention integrates the aforementioned mechanisms, and the final output is given by Equation 9:


$$A\,A\,\left(X\,\left(i,j\right)\right)=\left(A_{\rho \,\left(i,j\right)}+{\hat{Q}}_{\left(i,j\right)}\,T\right)\,V_{\rho \,\left(i,j\right)}+A_{\sigma \,\left(i,j\right)}\,V_{\sigma \,\left(X\right)}$$ (9)

## 4 Experiments and results analysis

### 4.1 Dataset and data preprocessing

In this study, a multitask brain tumor experiment was conducted using two publicly available datasets: the Brain tumor dataset released by Cheng et al. (2015) and the BRISC 2025 dataset introduced by Fateh et al. (2026). The Cheng dataset consists of 3,064 brain MRI images from patients, encompassing three types of brain tumors (denoted as Glioma: G, Meningioma: M, and Pituitary: P). For the BRISC 2025 dataset, the segmentation task subset was adopted, which includes 4,793 original brain MRI images along with their corresponding 4,793 binary mask annotations, also covering the same three tumor types (denoted as Glioma: G, Meningioma: M, and Pituitary: P). Both datasets provide pixel-level ground-truth masks for segmentation. The Brain Tumor dataset contains 3,064 MRI images with corresponding tumor region masks, while the BRISC 2025 segmentation subset contains 4,793 MRI images and 4,793 binary masks. Detailed information is presented in Table 1.

**TABLE 1:** Dataset information.

Dataset	Type	Images	Masks	Training	Testing	Total number
Brain tumor dataset	Glioma	1426	1426	1140	286	3064
	Meningioma	708	708	566	142	
	Pituitary	930	930	744	186	
BRISC 2025 dataset	Glioma	1401	1401	1147	254	4793
	Meningioma	1635	1635	1329	306	
	Pituitary	1757	1757	1457	300	

Both the Brain tumor dataset and the BRISC 2025 dataset consist of T1-weighted contrast-enhanced brain MRI images. During the preprocessing stage, the images were resized to 224 × 224 pixels, and the single channel was replicated and expanded to three channels to meet the input requirements of the model, followed by pixel normalization to eliminate variations in brightness and contrast. To address the issue of limited data scale and mitigate overfitting, data augmentation strategies such as random vertical and horizontal flipping and random intensity shift were employed to enhance data diversity while preserving image semantics. Finally, the dataset was randomly divided into mutually exclusive training and testing sets at a ratio of 8:2. Data augmentation was performed online only on the training set during each epoch, using random vertical and horizontal flipping and random intensity shift. The test set was not augmented. Since the augmentation is applied online, it does not increase the number of stored samples. Specifically, the Brain Tumor dataset contains 3,064 original images, with 2,450 images for training and 614 images for testing. The BRISC 2025 dataset contains 4,793 images and 4,793 corresponding masks, with 3,933 images and 3,933 corresponding masks for training, and 860 images and 860 corresponding masks for testing. The number of training samples per epoch remains 2,450 and 3,933, respectively, while the test sets remain unchanged at 614 and 860. Although the number of stored images remains unchanged, online augmentation dynamically generates different augmented versions in each epoch, thereby increasing the diversity of training samples without creating additional image files.

### 4.2 Loss function

Since UAC-Net jointly performs brain tumor classification and segmentation, its optimization objective comprises task-specific classification and segmentation losses. For the classification branch, the cross-entropy loss is employed to measure the discrepancy between the predicted class probabilities and the ground-truth labels. It is defined in Equation 10:


$$L_{c\,l\,s}=-\sum_{c=1}^{C}y_{c}\,\log\left(p_{c}\right)$$ (10)

where *C* denotes the number of tumor categories, *y*_*c*_ represents the ground-truth label of class *c* and *p*_*c*_ denotes the predicted probability for the corresponding class.

For the segmentation branch, a hybrid BCE-Dice loss is adopted. The binary cross-entropy (BCE) loss provides pixel-wise supervision by penalizing the discrepancy between the predicted segmentation probabilities and the ground-truth mask, and is defined in Equation 11:


$$L_{B\,C\,E}=-\frac{1}{N}\,\sum_{i=1}^{N}\left[y_{i}\,\log\left(p_{i}\right)+\left(1-y_{i}\right)\,\log\left(1-p_{i}\right)\right]$$ (11)

where *N* denotes the number of pixels, *y*_*i*_ϵ{0,1} is the ground-truth label of the *i*-th pixel, and *p*_*i*_ represents the predicted probability that the corresponding pixel belongs to the tumor region.

To further optimize the overlap between the predicted segmentation and the ground-truth mask, the Dice loss is introduced and defined in Equation 12:


$$L_{D\,i\,c\,e}=1-\frac{2\,\sum_{i=1}^{N}p_{i}\,y_{i}+\varepsilon }{\sum_{i=1}^{N}p_{i}+\sum_{i=1}^{N}y_{i}+\varepsilon }$$ (12)

where ε is a small smoothing constant introduced to avoid numerical instability. The Dice loss directly measures the regional overlap between the predicted and ground-truth masks and is particularly useful for brain tumor segmentation, where the lesion region may occupy only a small portion of the image.

The segmentation loss is therefore formulated as a weighted combination of BCE loss and Dice loss, as shown in Equation 13:


$$L_{s\,e\,g}=\alpha \,L_{B\,C\,E}+\beta \,L_{D\,i\,c\,e}$$ (13)

where α and β denote the weighting coefficients of the BCE and Dice losses, respectively.

Finally, the classification and segmentation objectives are jointly optimized through a weighted multi-task loss, as shown in Equation 14:


$$L_{t\,o\,t\,a\,l}=\lambda_{s\,e\,g}\,L_{s\,e\,g}+\lambda_{c\,l\,s}\,L_{c\,l\,s}$$ (14)

where λ_*seg*_ and λ_*cls*_ control the relative contributions of the segmentation and classification tasks, respectively. The initial values of λ_*seg*_ and λ_*cls*_ are set to 2 and 1, respectively. During training, these weighting coefficients are automatically updated according to the validation performance of the segmentation and classification tasks. By jointly optimizing the pixel-level segmentation objective and the image-level classification objective, the model can simultaneously learn lesion structural information and tumor category semantics, thereby facilitating complementary optimization between the two tasks.

### 4.3 Model parameters and hardware environment

All experiments were conducted on a single workstation running the Windows 10 operating system, equipped with 32 GB of RAM and an NVIDIA RTX 4060 Ti GPU with 16 GB of memory. The proposed model was implemented using the PyTorch deep learning framework. The Adam optimizer was adopted for model training, with an initial learning rate set to 0.0001. The batch size was set to 8, and the maximum number of training epochs was set to 160. A ReduceLROnPlateau learning-rate scheduler was adopted with a reduction factor of 0.2 and a patience of 5 epochs. An early stopping strategy was introduced to mitigate overfitting.

### 4.4 Evaluation metrics

For the image classification task, accuracy (Acc), recall (Re), precision (Pre), and F1-score (F1) were adopted as evaluation metrics. The formulas for these classification metrics are given in Equations 15–18:


$$R\,e\,c\,a\,l\,l=\frac{T\,P}{T\,P+F\,N}$$ (15)

$$\mathrm{Pr}e\,c\,i\,s\,i\,o\,n=\frac{T\,P}{T\,P+F\,P}$$ (16)

$$F\,1-s\,c\,o\,r\,e=\frac{2\,T\,P}{2\,T\,P+F\,P+F\,N}$$ (17)

$$\mathrm{Accuracy}=\frac{T\,N+T\,P}{T\,N+T\,P+F\,N+F\,P}$$ (18)

where the notations are TP = true positive, FP = false positive, TN = true negative, and FN = false negative.

For the image segmentation task, the evaluation metrics adopted include the Dice coefficient (Dice), sensitivity (Se, also known as recall) and IoU. The formulas for the Dice coefficient and IoU are given in Equations 19, 20:


$$D\,i\,c\,e=\frac{2\,T\,P}{2\,T\,P+F\,P+F\,N}$$ (19)

$$I\,o\,U=\frac{T\,P}{T\,P+F\,P+F\,N}$$ (20)

The 95% Hausdorff distance (HD95) is used to evaluate the boundary discrepancy between the predicted segmentation and the ground-truth mask. Instead of using the maximum boundary distance, HD95 is defined as the 95th percentile of the bidirectional distances between the predicted and ground-truth boundary point sets, which reduces the influence of extreme outliers. A smaller HD95 value indicates better agreement between the predicted boundary and the ground truth. The HD95 metric is calculated as shown in Equation 21:


H⁢D⁢95⁢(L,P)=max⁡{P⁢95⁢(d⁢(L,P)),P⁢95⁢(d⁢(P,L))} (21)

where *L* and *P* denote the ground truth and predicted boundary point sets, respectively. *d(L,P)* and *d(P,L)* represent the directed point-to-set distance distributions, and *P95*(.) denotes the 95th percentile.

### 4.5 Experimental results

Table 2 presents the classification and segmentation performance of UAC-Net on the two datasets. On the Brain Tumor dataset, UAC-Net achieved average precision, recall, and F1-score values of 98.47%, 98.41%, and 98.44%, respectively, with an overall classification accuracy of 98.53%. For the segmentation task, the Dice coefficient, sensitivity, IoU, and HD95 were 81.39%, 83.61%, 72.66%, and 5.782 mm, respectively. On the BRISC 2025 dataset, UAC-Net achieved better overall performance, with average precision, recall, and F1-score values of 99.08%, 99.04%, and 99.06%, respectively, and an overall classification accuracy of 99.07%. For segmentation, the Dice coefficient, sensitivity, IoU, and HD95 reached 86.61%, 88.41%, 79.63%, and 4.713 mm, respectively. Overall, these results demonstrate that UAC-Net achieved favorable performance in both classification and segmentation tasks on the two datasets.

**TABLE 2:** Experimental results for model UAC-Net.

Type	Classification				Segmentation			
	Pre (%)	Re (%)	F1 (%)	Acc (%)	Dice (%)	Se (%)	IoU (%)	HD95 (mm)
Brain tumor dataset								
Glioma	98.60	98.60	98.60	98.53	81.39	83.61	72.66	5.782
Meningioma	97.87	97.18	97.52					
Pituitary	98.93	99.46	99.19					
Average	98.47	98.41	98.44					
BRISC 2025 dataset								
Glioma	99.21	98.43	98.81	99.07	86.61	88.41	79.63	4.713
Meningioma	98.38	99.02	98.70					
Pituitary	99.67	99.67	99.67					
Average	99.08	99.04	99.06					

Table 3 reports the computational complexity and runtime of UAC-Net. The model contains 74.97 M parameters and requires 36.52 GFLOPs, which is moderate for a multi-task network. All experiments were conducted on a single workstation with an NVIDIA RTX 4060 Ti GPU (16 GB) and 32 GB RAM. On the Brain tumor dataset, training takes 1.34 h and testing takes 19 s. On the larger BRISC 2025 dataset, training and testing times are 2.14 h and 30 s, respectively. The model is trained with a batch size of 8 for 160 epochs on a single consumer-grade GPU, indicating low resource requirements. These results suggest that UAC-Net has moderate computational complexity and fast inference, supporting its potential for clinical application.

**TABLE 3:** Model complexity and runtime on brain tumor and BRISC 2025 datasets.

Dataset	Params (M)	FLOPs (G)	Train time (h)	Test time (s)
Brain tumor dataset	74.97	36.52	1.34	19
BRISC 2025 dataset	74.97	36.52	2.14	30

Grad-CAM++ generates class-discriminative heatmaps by leveraging the gradient information flowing into the final convolutional layer, providing an intuitive visualization of the input regions that are most influential to the model’s decision (Chattopadhay et al., 2018). In this study, we applied Grad-CAM++ to the classification branch to visually interpret the model’s focus. Figure 4 presents the resulting heatmaps superimposed on representative tumor images. It can be observed that the high-activation (i.e., “hot”) regions predominantly overlap with tumor-related areas and align closely with the ground-truth lesion locations. This consistency indicates that the classification branches based on the UAC-Net model are capable of learning clinically significant distinguishing features, rather than relying on spurious correlations or background artifacts.

**FIGURE 4:**
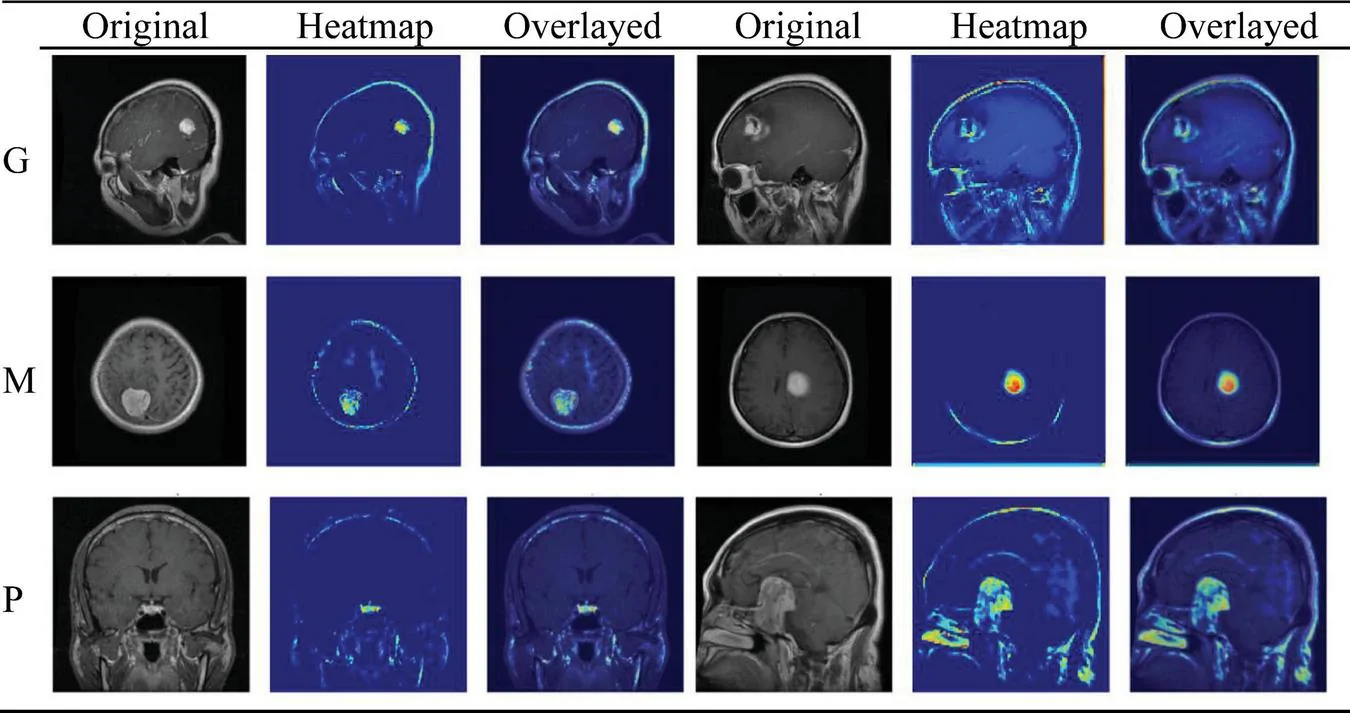
Grad-CAM++ visualization results of UAC-Net (Brain tumor dataset).

These learned features not only contribute to accurate tumor classification but also effectively guide the segmentation of tumor region, as illustrated in Figure 5. The above results demonstrate that the proposed UAC-Net not only achieves high-precision classification but also possesses strong spatial localization capability, validating its effectiveness and interpretability in computer-aided diagnosis of brain tumors.

**FIGURE 5:**
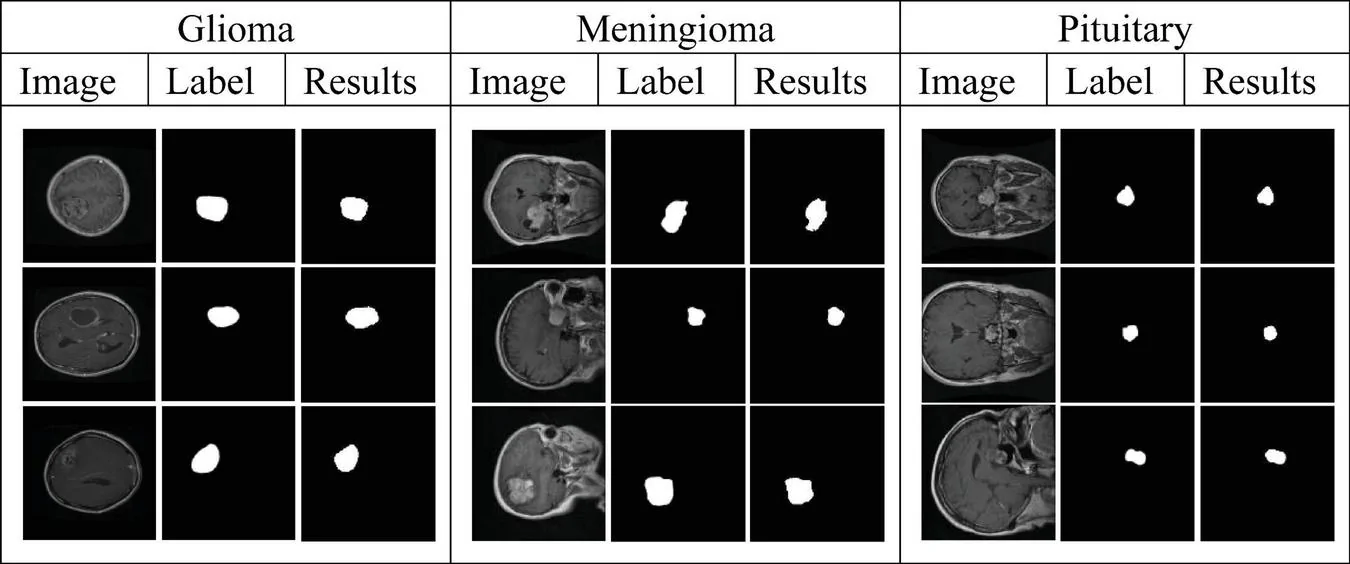
Segmentation results of UAC-Net on different tumor types (Brain tumor dataset).

To evaluate the reproducibility of the proposed UAC-Net, five independent repeated runs were performed on the Brain Tumor dataset under identical preprocessing, augmentation, and training settings. Table 4 reports the mean and standard deviation (SD) of the main evaluation metrics. UAC-Net achieved an accuracy of 98.42 ± 0.22%, a Dice score of 81.50 ± 0.57%, an IoU of 72.11 ± 0.56%, and an HD95 of 5.73 ± 0.46 mm. The small SDs indicate that the performance did not depend on a single favorable run. These mean values are consistent with the representative result in Table 2, where UAC-Net obtained 98.53% accuracy, 81.39% Dice, 72.66% IoU, and 5.782 mm HD95 on the same dataset, with all differences within one SD. Across the five runs, accuracy ranged from 98.21% to 98.72%, Dice from 80.92% to 82.35%, IoU from 71.24% to 72.78%, and HD95 from 5.22 to 6.28 mm. These variations are modest and do not change the overall performance trend or the main conclusions. Overall, these repeated-run results confirm the reproducibility of UAC-Net and further support the reliability of the comparative and ablation.

**TABLE 4:** Results of 5 repeated runs (Brain tumor dataset).

Number of times	Acc (%)	Dice (%)	IoU (%)	HD95 (mm)
Run1	98.23	82.35	72.78	5.22
Run2	98.37	81.15	72.28	5.47
Run3	98.21	81.78	72.01	5.52
Run4	98.56	80.92	72.24	6.14
Run5	98.72	81.30	71.24	6.28
**Mean+std**	**98.42 ± 0.22**	**81.50 ± 0.57**	**72.11 ± 0.56**	**5.73 ± 0.46**

Bold values indicate the mean ± standard deviation across five independent runs.

### 4.6 Comparison of experiments with different framework designs

To validate the effectiveness and superiority of the proposed UAC-Net, we conducted comparative experiments with several mainstream medical image methods, including U-Net, Attention U-Net, SwinUNet, and TransUNet. All compared models were trained and tested on exactly the same datasets and data splits as UAC-Net, with identical preprocessing, augmentation, and training settings. For segmentation-only models, a classification head identical to that of UAC-Net was added to their encoders to enable a fair multi-task comparison.

As shown in Table 5, comparative experiments were conducted on the Brain tumor dataset and the BRISC 2025 dataset to evaluate different network models. On the Brain tumor dataset, the classic U-Net achieved a classification accuracy of 97.39% and a Dice coefficient of 78.56%. By introducing attention gates to suppress irrelevant background regions and emphasize lesion-related features, Attention U-Net improved the classification accuracy and Dice coefficient to 98.53% and 79.55%, respectively. In contrast, SwinUNet and TransUNet obtained Dice coefficients of 70.57% and 76.33%, respectively, which were lower than those of the convolution-based models. This suggests that, on this dataset, Transformer-based and CNN-Transformer hybrid architectures still have certain limitations in modeling small and irregular lesion regions in small-sample brain tumor MRI images. The proposed UAC-Net achieved the best overall performance on this dataset, with a classification accuracy, Dice coefficient, IoU, and HD95 of 98.53%, 81.39%, 72.66%, and 5.782, respectively, demonstrating its superior performance in classification, region overlap, and boundary localization.

**TABLE 5:** Experimental results for different network models.

Model	Classification				Segmentation			
	Pre (%)	Re (%)	F1 (%)	Acc (%)	Dice (%)	Se (%)	IoU (%)	HD95 (mm)
Brain tumor dataset								
UNet (Ronneberger et al., 2015)	97.07	96.94	97.00	97.39	78.56	80.82	69.42	7.731
Attention-UNet (Schlemper et al., 2019)	98.47	**98.42**	98.44	98.53	79.55	80.88	70.68	6.914
SwinUNet (Cao et al., 2023)	93.75	93.31	93.52	94.14	70.57	74.87	59.42	8.514
TransUNet (Chen et al., 2024)	95.71	96.24	95.96	96.42	76.33	77.65	66.77	9.168
UAC-Net	**98.47**	98.41	**98.44**	**98.53**	**81.39**	**83.61**	**72.66**	**5.782**
BRISC 2025 dataset								
UNet (Ronneberger et al., 2015)	98.97	98.91	98.94	98.95	85.91	87.68	78.82	5.107
Attention-UNet (Schlemper et al., 2019)	98.24	98.33	98.28	98.26	82.77	86.80	74.99	6.713
SwinUNet (Cao et al., 2023)	97.88	97.62	97.73	97.79	79.81	81.87	70.51	5.947
TransUNet (Chen et al., 2024)	98.21	98.05	98.13	98.14	84.96	86.34	77.54	6.265
UAC-Net	**99.08**	**99.04**	**99.06**	**99.07**	**86.61**	**88.41**	**79.63**	**4.713**

Bold values indicate the best performance for each metric (higher is better, except for HD95, where lower is better).

On the BRISC 2025 dataset, U-Net provided a strong baseline, achieving a classification accuracy of 98.95% and a Dice coefficient of 85.91%. However, Attention U-Net, SwinUNet, and TransUNet performed worse than U-Net in both classification accuracy and Dice coefficient, suggesting that more complex attention- or Transformer-based architectures did not necessarily provide stable improvements over the convolutional baseline. Compared with these models, UAC-Net achieved the best results, with a classification accuracy, Dice coefficient, sensitivity, IoU, and HD95 of 99.07%, 86.61%, 88.41%, 79.63%, and 4.713, respectively. Compared with the strong U-Net baseline, UAC-Net improved the Dice coefficient and IoU by 0.70 and 0.81 percentage points, respectively, while reducing HD95 by 0.394. These results indicate that the proposed model not only maintains high classification accuracy but also provides more stable advantages in tumor region segmentation and boundary error control. Overall, UAC-Net demonstrates favorable classification and segmentation performance on both datasets, validating its effectiveness and generalization capability for multi-task brain tumor MRI image analysis.

As shown in Figure 6, the accuracy and loss curves of different models during the validation process are plotted to evaluate their performance. From the validation accuracy curves, it can be observed that UAC-Net achieves the fastest convergence and attains the highest final accuracy among all compared models. From the validation loss curves, UAC-Net exhibits the most stable decline in loss values and converges to the lowest loss level, demonstrating superior optimization stability and generalization capability. In contrast, the other models exhibit varying degrees of fluctuation or slow convergence during training. These experimental results demonstrate that the proposed method consistently outperforms various baseline models in both classification accuracy and segmentation performance, fully validating its superiority in multi-task brain tumor analysis.

**FIGURE 6:**
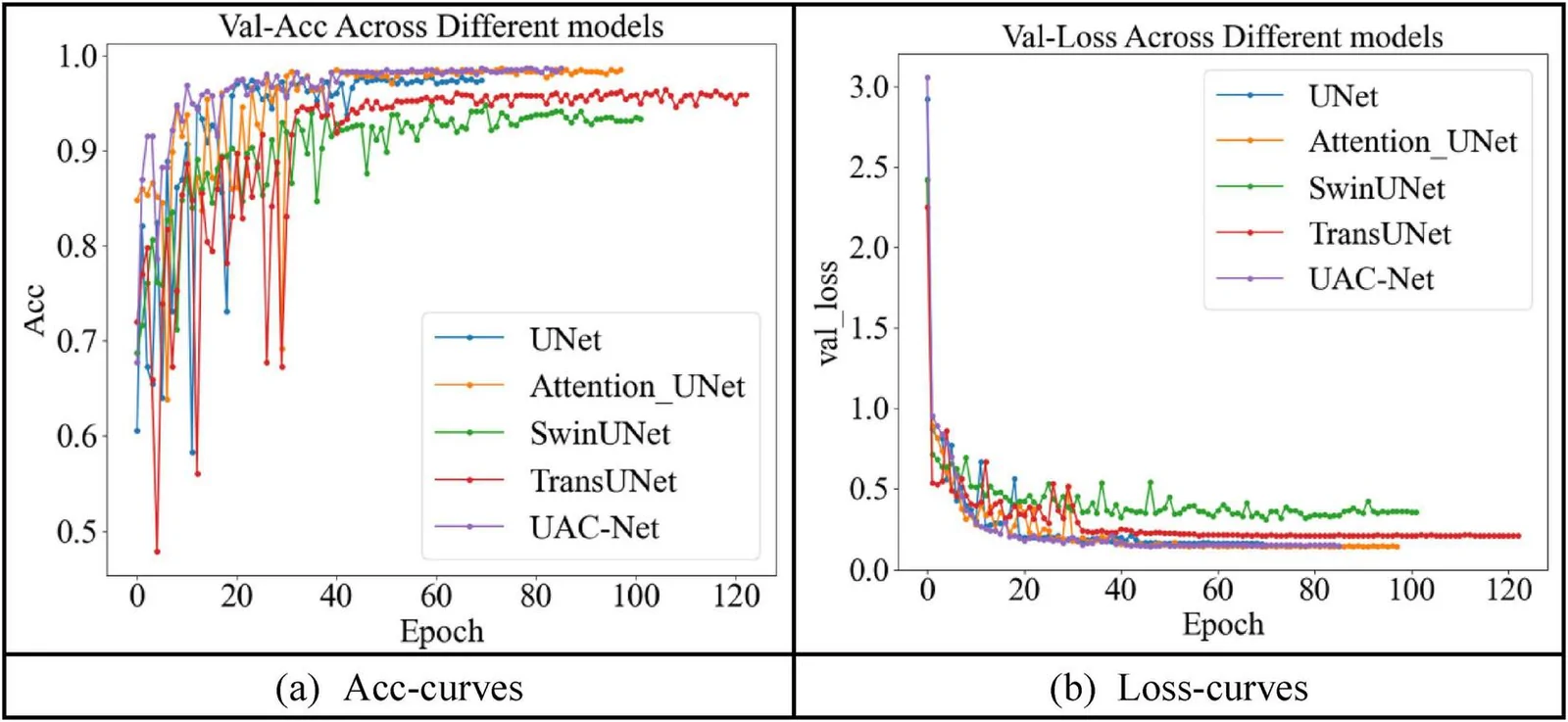
Comparison of accuracy and loss curves of different models during validation (Brain tumor dataset). **(a)** Accuracy curves. **(b)** Loss curves.

### 4.7 Ablation experiments

To systematically evaluate the individual contributions and synergistic effects of UAC-Net, we conducted ablation experiments by progressively integrating the ALMFF module and the ACA module into the baseline model. As presented in Table 6, the baseline model achieved a classification accuracy of 97.39% and a Dice coefficient of 78.56%. The introduction of the ACA module alone led to substantial improvements, raising the classification accuracy to 98.21% (a relative increase of 0.84%) and the Dice coefficient to 80.55% (a relative increase of 2.53%), while reducing the HD95 from 7.731 mm to 6.141 mm, highlighting the effectiveness of the ACA module in enhancing global contextual modeling. In contrast, when ALMFF was introduced alone, the classification accuracy slightly increased to 97.56%, whereas the Dice coefficient decreased from 78.56% to 76.45% and the HD95 increased to 9.478 mm. This result suggests that directly fusing adjacent-layer features may introduce cross-level feature interference or redundant background responses when no explicit contextual attention is used to regulate the fused representations.

**TABLE 6:** Results of ablation experiments of ALMFF, ACA (Brain tumor dataset).

Methods	Classification				Segmentation			
	Pre (%)	Re (%)	F1 (%)	Acc (%)	Dice (%)	Se (%)	IoU (%)	HD95 (mm)
Baseline	97.07	96.94	97.00	97.39	78.56	80.82	69.42	7.731
Baseline + ALMFF	97.19	97.24	97.21	97.56	76.45	78.24	67.50	9.478
Baseline + ACA	97.95	98.12	98.03	98.21	80.55	83.88	71.55	6.141
Baseline + ALMFF + ACA	98.47	98.41	98.44	98.53	81.39	83.61	72.66	5.782

However, when ALMFF was combined with ACA, the model achieved the best overall performance, with a classification accuracy of 98.53%, a Dice coefficient of 81.39%, an IoU of 72.66%, and an HD95 of 5.782 mm. This indicates that ACA plays a dominant role in suppressing irrelevant responses and enhancing lesion-related contextual information, while ALMFF provides complementary multi-scale structural cues after being guided by ACA. Therefore, the performance gain of UAC-Net is mainly attributed to the synergistic interaction between ACA and ALMFF rather than the independent effect of ALMFF alone.

Figure 7 illustrates the confusion matrices of the models under the ablation experimental setup. As observed from the confusion matrices, the baseline model exhibits varying degrees of misclassification among the three brain tumor types, with notable confusion particularly between meningioma and glioma. After introducing the ALMFF module alone, classification confusion is partially alleviated, yet the improvement remains limited. With the standalone introduction of the ACA module, the classification accuracy for each tumor type improves substantially, and the number of misclassified samples decreases significantly, indicating that the ACA module plays a dominant role in global semantic modeling. When both the ACA and ALMFF modules are simultaneously integrated, the confusion matrix achieves an optimal diagonal distribution, with nearly all samples correctly classified. It is worth noting that the Brain Tumor dataset exhibits a certain degree of class imbalance. The test set contains 286 glioma, 142 meningioma, and 186 pituitary samples, while the corresponding training set contains 1,140 glioma, 566 meningioma, and 744 pituitary samples, as listed in Table 1. From the confusion matrix in Figure 7, the per-class recall can be calculated as the ratio of the diagonal entry to the corresponding row sum. The resulting recalls are 98.60%, 97.18%, and 99.46% for glioma, meningioma, and pituitary, respectively. The macro-average recall (balanced accuracy) is 98.41% and the macro-average F1 is 98.44%, indicating that the model does not show obvious bias toward the majority class.

**FIGURE 7:**
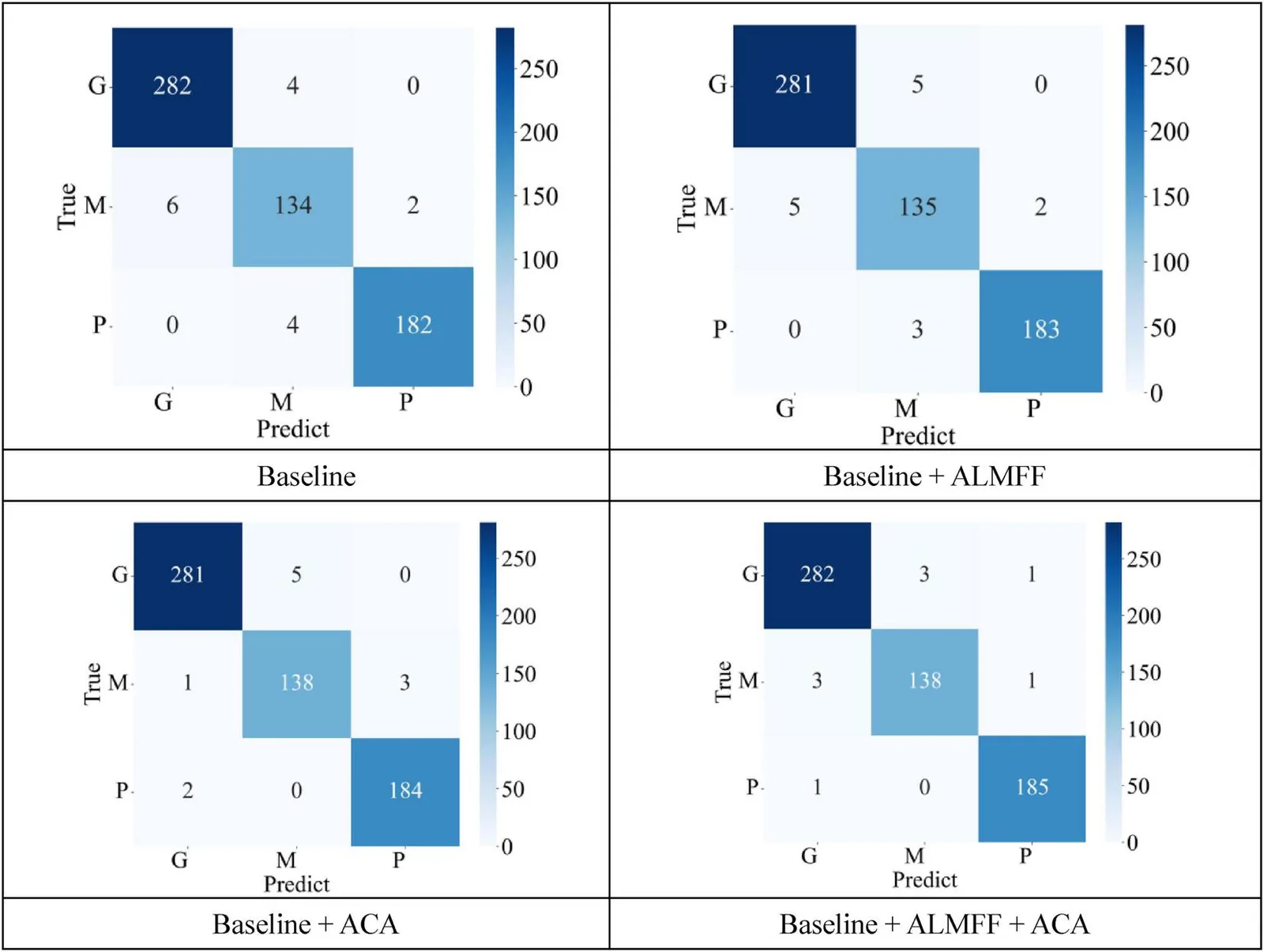
Confusion matrix comparison across ablation settings (Brain tumor dataset).

To validate the synergistic effect of multi-task learning, we conducted comparative experiments on the classification task, segmentation task, and joint multi-task, respectively, with the results presented in Table 7. The standalone classification model achieved a classification accuracy of 98.20%, while the standalone segmentation model attained a Dice coefficient of 72.93%. In contrast, the proposed multi-task learning model improved the classification accuracy to 98.53% (a relative increase of 0.34%) and substantially boosted the Dice coefficient to 81.39% (a relative increase of 11.60%). These results demonstrate that multi-task learning achieves complementary optimization between tasks through shared feature representation: the classification task provides global semantic constraints, while the segmentation task reinforces spatial structural information, with their synergistic effect jointly enhancing the overall performance of the model. The findings further confirm that the synergistic mechanism of multi-task learning substantially enhances the representational capacity and generalization performance of the model in multi-task brain tumor classification and segmentation.

**TABLE 7:** Results of ablation experiments on model structures (Brain tumor dataset).

Methods	Acc (%)	Dice (%)
(1) Class task	98.20	–
(2) Segmentation task	–	72.93
(3) multi-task	98.53	81.39

To further investigate how ALMFF and ACA affect lesion localization and feature representation, qualitative visualization experiments were conducted under different ablation settings, as shown in Figure 8. The first three rows present representative segmentation prediction–ground truth overlays, while the last row shows the corresponding classification Grad-CAM++ visualizations. Compared with the baseline, introducing ALMFF modifies the predicted lesion regions through cross-level feature interaction; however, its standalone use does not consistently improve boundary agreement, which is consistent with the quantitative ablation results in Table 6. In contrast, the incorporation of ACA generally improves the alignment between predicted and ground-truth lesion regions by enhancing lesion-related contextual representations. When ALMFF and ACA are jointly employed, the predicted regions exhibit more consistent overlap with the ground truth, while the Grad-CAM++ responses are more concentrated around tumor-related regions with reduced irrelevant background activation. These qualitative observations further support the complementary roles of ACA and ALMFF: ACA enhances lesion-aware contextual modeling, whereas ALMFF provides multi-scale structural information, and their combination leads to more reliable feature representation and prediction.

**FIGURE 8:**
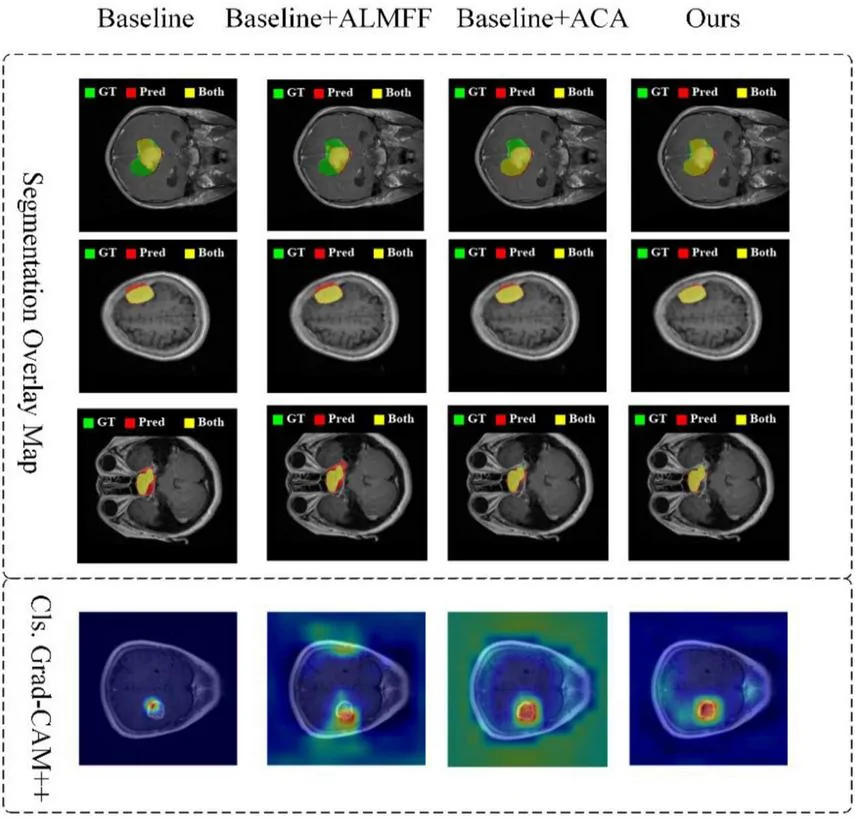
Ablation visualization of ALMFF and ACA.

To further validate the design of ACA and ALMFF, ablation experiments were conducted by replacing them with standard attention and fusion modules under identical training and testing settings.

Table 8 reports the ablation results with standard attention modules. ACA achieves the best overall performance, with the highest Dice (81.39%) and IoU (72.66%) and the lowest HD95 (5.782 mm). SE slightly improves over the baseline by recalibrating channel responses, but it does not expand the spatial receptive field, and its Dice gain remains limited (79.53% vs. 78.56%). CBAM degrades Dice to 75.72% and increases HD95 to 11.711 mm, indicating that its spatial attention is insufficient for irregular and boundary-ambiguous tumor regions under this setting. Transformer also underperforms the baseline, with Dice of 75.56% and HD95 of 18.547 mm, likely because its global self-attention loses local spatial details and is difficult to optimize on this small dataset. In contrast, ACA integrates sliding-window local attention with global downsampled attention, enabling each token to capture both local spatial details and long-range contextual dependencies.

**TABLE 8:** Ablation experiments with standard attention modules (Brain tumor dataset).

Methods	Acc (%)	Dice (%)	IoU (%)	HD95 (mm)
Baseline	97.39	78.56	69.42	7.731
Baseline+SE	97.88	79.53	70.94	7.279
Baseline+CBAM	98.20	75.72	66.56	11.711
Baseline+ Transformer	97.72	75.56	66.05	18.547
**Ours**	**98.53**	**81.39**	**72.66**	**5.782**

Bold values indicate the best performance for each evaluation metric. Higher values are better for Acc, Dice, and IoU, whereas lower values are better for HD95.

Table 9 compares different fusion mechanisms with ACA fixed. The simple concatenation baseline achieves an accuracy of 98.21%, a Dice score of 80.55%, and an HD95 of 6.141 mm, but lacks adaptive selection and may introduce redundant background responses from adjacent layers. Attention Gate improves the accuracy to 98.53% and the Dice score to 80.73%, yet increases HD95 to 7.463 mm, indicating that it reweights features without explicitly modeling multi-scale interaction. ASPP reduces the accuracy to 97.39% and the Dice score to 80.21%, and increases HD95 to 10.836 mm, suggesting that dilated convolutions may fail to preserve local spatial details and boundary-related information that are critical for small and irregular brain tumors. In contrast, ALMFF achieves the highest accuracy of 98.53%, the highest Dice score of 81.39%, the highest IoU of 72.66%, and the lowest HD95 of 5.782 mm. ALMFF first aligns adjacent-layer features via transposed convolution and then employs dual-branch 3 × 3 and 5 × 5 convolutions to capture local spatial details and boundary-related information as well as broader contextual cues. The resulting features are fused by element-wise multiplication to enhance consistent responses and suppress redundant background information, followed by a residual connection to stabilize feature propagation. These results demonstrate the effectiveness of ALMFF for cross-level multi-scale fusion.

**TABLE 9:** Ablation experiments with standard fusion modules (Brain tumor dataset).

Methods	Acc (%)	Dice (%)	IoU (%)	HD95 (mm)
Baseline+ACA+concat	98.21	80.55	71.55	6.141
Baseline+ACA+Attention Gate	**98.53**	80.73	72.03	7.463
Baseline+ACA+ASPP	97.39	80.21	71.50	10.836
**Ours**	**98.53**	**81.39**	**72.66**	**5.782**

Bold values indicate the best performance for each evaluation metric. Higher values indicate better performance for Acc, Dice, and IoU, whereas lower values indicate better performance for HD95.

These ablation experiments, together with the ablation study in Table 6, indicate that ACA and ALMFF are better suited for multi-scale contextual modeling in brain tumor MRI than standard attention and fusion mechanisms.

## 5 Discussion

UAC-Net achieves superior results compared to the other models in both classification and segmentation tasks. The classification performance benefits from multi-task collaborative optimization: the shared encoder enables segmentation features to assist in classification localization, the ACA module enhances global perception, and GAP improves feature robustness. The segmentation performance is attributed to the ACA module, which integrates local and global attention, and the ALMFF module, which facilitates interaction between adjacent layer features, collectively enhancing the representation capability for small-scale tumors and the accuracy of segmentation boundaries. It should be noted that ALMFF alone does not consistently improve segmentation performance, probably because adjacent-layer fusion may also introduce redundant or noisy low-level responses. Its effectiveness becomes more evident when coupled with ACA, which provides contextual guidance to suppress irrelevant activations and enhance lesion-related fused features.

To comprehensively evaluate the classification performance of the proposed UAC-Net model on the brain tumor MRI dataset, we compared it with several existing methods on the same dataset. Table 10 presents the comparison results between the proposed method and existing methods on the brain tumor classification task. As shown in the table, UAC-Net achieves the best performance across all evaluation metrics. Notably, compared with the best-performing existing method, UAC-Net improves the classification accuracy from 98.05% to 98.53%, corresponding to an increase of 0.48 percentage points, while also achieving a higher F1-score. These results fully validate the effectiveness and superiority of the proposed model in brain tumor classification tasks.

**TABLE 10:** Comparison of brain tumor classification with existing methods’performance (Brain tumor dataset).

Model	Pre (%)	Re (%)	F1 (%)	Acc (%)
Huang et al., 2026	97.93	97.59	97.75	98.04
Fan et al., 2026	97.76	–	97.79	98.05
Huang et al., 2025	95.17	95.78	95.46	95.88
Li and Zhou, 2025	97.80	98.20	98.00	98.04
**Ours**	98.47	98.41	98.44	98.53

Table 11 presents the performance comparison between the proposed model and existing methods on the brain tumor segmentation task. Kasar et al. (2024) and Koleilat et al. (2025) achieved mean Dice coefficients of 80.01% and 80.03%, respectively. In contrast, the proposed UAC-Net model attained a mean Dice coefficient of 81.39%, outperforming the above two methods by 1.38 and 1.36 percentage points, respectively. This result demonstrates that UAC-Net surpasses existing methods in segmentation accuracy, further validating its effectiveness and superiority in brain tumor segmentation tasks.

**TABLE 11:** Comparison of brain tumor segmentation with existing methods’performance (Brain tumor dataset).

Models	Mean Dice (%)
Kasar et al., 2024	80.01
Koleilat et al., 2025	80.03
Ours	81.39

Despite the favorable overall performance, several limitations of UAC-Net should be acknowledged. First, the segmentation of very small tumors remains challenging because such lesions occupy only a limited number of pixels, and fine-grained spatial information may be weakened during repeated downsampling. Second, when the lesion-to-background contrast is weak or tumor boundaries are ambiguous, the available local appearance cues may be insufficient for precise delineation, potentially resulting in under-segmentation or over-segmentation around the tumor margins. In addition, tumors with highly irregular morphology may still lead to boundary deviations between the predicted masks and the ground-truth annotations, which is also reflected by the remaining HD95 errors. The qualitative and ablation results further indicate that cross-level feature fusion does not always improve boundary agreement when used independently, as redundant background responses or cross-level feature interference may be introduced. Although ACA can alleviate these effects by enhancing lesion-related contextual representations, challenging cases with small lesions, weak contrast, or indistinct boundaries cannot be completely resolved. Future work will therefore focus on several directions. Boundary-aware learning and uncertainty modeling will be further investigated to improve robustness for these challenging tumor cases. Semi-supervised learning could also be explored to utilize unlabeled or partially labeled MRI data and reduce the dependence on pixel-level annotations. In addition, the current framework could be extended to multi-modal MRI by integrating complementary information from different imaging sequences. Finally, model compression strategies, such as lightweight network design, knowledge distillation, pruning, and quantization, could be investigated to further reduce computational and memory requirements and facilitate practical deployment.

Beyond these methodological extensions, the clinical translation of UAC-Net also requires consideration of its integration into real-world MRI workflows. UAC-Net could be integrated into the MRI analysis workflow as a computer-aided decision-support tool rather than a replacement for radiologists. After standard image preprocessing, the framework can simultaneously provide tumor-type predictions and segmentation maps, allowing clinicians to review the predicted category and lesion extent and, when necessary, manually correct the segmentation results before further diagnostic or treatment-planning decisions. Nevertheless, several challenges remain before real-world clinical deployment. First, despite the use of intensity normalization and data augmentation, clinical MRI data may exhibit substantial variations across scanners, institutions, acquisition protocols, spatial resolutions, and image quality, which may cause domain shifts and affect model robustness. Second, UAC-Net demonstrated consistent performance on two independent publicly available datasets; however, further validation on multi-institutional cohorts with greater variations in scanners, acquisition protocols, patient populations, and tumor types would further strengthen the evaluation of its robustness and generalizability. Third, annotation variability among radiologists, particularly for low-contrast or boundary-ambiguous lesions, may introduce uncertainty into pixel-level reference labels. Future studies should therefore include multicenter and external validation, image harmonization or domain-adaptation strategies, and multiple-expert annotations or consensus labeling. Finally, model calibration, computational efficiency, integration with clinical imaging systems, and prospective clinical evaluation should be further investigated to facilitate practical deployment.

## 6 Conclusion

This paper proposes UAC-Net, a unified multi-task learning framework for joint brain tumor classification and segmentation from MRI images. Built upon a shared encoder, UAC-Net incorporates the ACA module to enhance local and global contextual modeling and the ALMFF module to strengthen cross-level multi-scale feature interaction. Through collaborative optimization of the classification and segmentation tasks, the framework effectively exploits the complementarity between image-level semantic information and pixel-level structural information. Experimental results on the Brain Tumor and BRISC 2025 datasets demonstrate the effectiveness of the proposed method, achieving classification accuracies of 98.53% and 99.07% and Dice coefficients of 81.39% and 86.61%, respectively. The comparative and ablation experiments further validate the contributions of ACA and ALMFF to improving lesion representation and multi-task performance. Overall, UAC-Net provides an effective unified framework for brain tumor MRI classification and segmentation.

## Data Availability

The original contributions presented in this study are included in this article/Supplementary material, further inquiries can be directed to the corresponding author.
